# Population prevalence of edentulism and its association with depression and self-rated health

**DOI:** 10.1038/srep37083

**Published:** 2016-11-17

**Authors:** Stefanos Tyrovolas, Ai Koyanagi, Demosthenes B. Panagiotakos, Josep Maria Haro, Nicholas J. Kassebaum, Vanessa Chrepa, Georgios A. Kotsakis

**Affiliations:** 1 Parc Sanitari Sant Joan de Déu, Universitat de Barcelona. Fundació Sant Joan de Déu, Dr Antoni Pujadas, 42, Sant Boi de Llobregat, 08830, Barcelona, Spain; 2 Instituto de Salud Carlos III, Centro de Investigación Biomédica en Red de Salud Mental, CIBERSAM, Monforte de Lemos 3-5, Pabellón 11, 28029, Madrid, Spain; 3 Department of Nutrition and Dietetics, School of Health Science and Education, Harokopio University, Athens, Greece; 4 Institute for Health Metrics and Evaluation & Department of Anesthesiology & Pain Medicine, Seattle Children’s Hospital, University of Washington, Seattle, WA, USA; 5 Department of Endodontics, University of Texas Health Science Center at San Antonio, San Antonio, Texas, USA; 6 Department of Periodontics, University of Washington, Seattle, WA, USA

## Abstract

Edentulism is associated with various adverse health outcomes but treatment options in low- and middle-income countries (LMICs) are limited. Data on its prevalence and its effect on mental health and overall-health is lacking, especially from LMICs. Self-reported data on complete edentulism obtained by standardized questionnaires on 201,953 adults aged ≥18 years from 50 countries which participated in the World Health Survey (WHS) 2002–2004 were analyzed. Age and sex-standarized edentulism prevalence ranged from 0.1% (95% CI = 0.0–0.3) (Myanmar) to 14.5% (95% CI = 13.1–15.9) (Zimbabwe), and 2.1% (95% CI = 1.5–3.0) (Ghana) to 32.3% (95% CI = 29.0–35.8) (Brazil) in the younger and older age groups respectively. Edentulism was significantly associated with depression (OR 1.57, 95% CI = 1.23–2.00) and poor self-rated health (OR 1.38, 95% CI = 1.03–1.83) in the younger group with no significant associations in the older age group. Our findings highlight the edentulism-related health loss in younger persons from LMICs. The relative burden of edentulism is likely to grow as populations age and live longer. Given its life-long nature and common risk factors with other NCDs, edentulism surveillance and prevention should be an integral part of the global agenda of NCD control.

In 2013, oral disorders caused 16.5 million years lived with disability (YLDs) worldwide with edentulism accounting for one third of the burden^1^. Edentulism is defined as the loss of all natural teeth and is an important public health issue globally for its high prevalence (exceeding 10% in adults aged ≥ 50 years) and associated disability^2^,^3^. Notably, edentulism is also observed in young age groups with this condition being more prevalent in the socially deprived^4^. Although it is not fatal, edentulism directly affects facial appearance, nutrition, and the ability to eat, speak, and socialize. In contrast to other chronic morbid conditions that may be amenable to therapeutic treatment, edentulism is a definitive condition that is the endpoint of periodontal disease and dental caries. Dental caries are considered to be the main cause of edentulism in ages <45 years, while periodontal disease is the primary cause of tooth loss in older ages^5^. Being a definitive condition, edentulism reflects a life-long steady state of disability that is expected to carry its burden for the duration of a person’s life^6^.

Over the last 20 years, the global burden of edentulism on disability has declined on average. However, in contrast to high-income countries (HICs) where the prevalence of edentulism is decreasing, an opposite trend is observed in low- and middle-income countries (LMICs) where the rate of edentulism is increasing, mainly as the result of increments in periodontal diseases and caries^7^. The increase of edentulism concomitant with the consistent increase in average life expectancy in LMICs significantly adds to the rampant burden of chronic non-communicable diseases (NCDs) that are now the leading causes of morbidity and mortality in most countries outside sub-Saharan Africa^8^. This epidemiological transition has refocused the attention of global health policy organizations to the prevention and control of NCDs.

Edentulism shares the same cluster of known modifiable risk factors as other NCDs such as smoking, alcohol use, and unhealthy carbohydrate-rich dietary behaviors^3^ - conditions highly prevalent or increasing at rapid rates in LMICs^9^. Apart from these individual factors, edentulism is also strongly associated with lack or limited access to dental care and fluoridated water and higher income inequalities, which are conditions more common in LMICs. For example, in many resource-limited settings, tooth extraction is the only cure available for dental problems; a practice that leads to early tooth loss^10^. Furthermore, given that secondary restorative dental care that is necessary to rehabilitate edentulism with functional and esthetic prostheses is sub-optimal or not affordable in many LMICs, edentulism may have a pronounced negative effect on mental health and psychological well-being of individuals in resource-limited settings.

To date, despite the potentially profound negative effect of edentulism on population’s health status and wellbeing^11^, the epidemiology of edentulism is not fully understood and data on the correlates and adverse health outcomes from LMICs are scarce^3^. The World Health Survey 2002–2004 (WHS) provides a unique opportunity to assess the prevalence and risk factors of edentulism and its effect on overall health and well-being in mostly nationally representative samples of the adult population from numerous LMICs. In the present analysis, we aimed to: (1) estimate the prevalence of edentulism in WHS countries, (2) investigate the correlates of edentulism, and (3) assess how edentulism is associated with depression and decrement in self-rated health in LMICs.

## Methods

The WHS was a cross-sectional study that was conducted between 2002 and 2004 in 70 countries that represented all regions of the world. The aim of the WHS was to collect global comparable population data on health and wellbeing among adults. Extensive details of the survey have been provided elsewhere (http://www.who.int/healthinfo/survey/en/).

### Sampling strategy

Single-stage random sampling and stratified multi-stage random cluster sampling were conducted in 10 and 60 countries respectively. Data were nationally representative in all countries except China, Comoros, the Republic of Congo, Ivory Coast, India, and Russia. Each member of the household had equal probability of being selected with the use of Kish tables^12^. The same questionnaire was used in all locations, although some used a shorter version. Questionnaires were back- and forward-translated in multiple languages to maximize comparability using a standard WHO linguistic protocol. Face-to-face interviews were used everywhere except Luxembourg and Israel where data collection occurred via telephone by trained interviewers. The interviewers participated in a training course for over a week and prior to collecting data they also participated in supervised field interviews^12^. The individual response rate across all countries was 98.5%^13^. Sampling weights were generated to adjust for non-response and for the population distribution reported by the United Nations Statistical Division^12^. Ethical approval for the WHS was obtained from the WHO Ethical Review Committee and local ethics research review boards. All WHS methodology was carried out in “accordance” with the approved guidelines from the WHO Ethical Review Committee. Participants were informed about the aims and procedures of the study and gave their consent prior to being interviewed.

### Variables

*Socio-demographic variables* included age, sex, wealth, and education. Country-specific wealth quintiles were generated using principal component analysis of 15–20 assets. Education was categorized in four groups: no formal education, primary education, secondary or high school completed, or tertiary education completed. *Edentulism* was assessed by the question “Have you lost all your natural teeth?” Those who responded affirmatively were assumed to have edentulism. *Depression* was defined as either or both: (1) past 12-month depression based on duration and persistence of depressive symptoms using the DSM-IV algorithm^14^; and (2) self-reported lifetime depression diagnosis. Details of the diagnosis based on past 12-month symptoms are provided in Appendix 1. *Self-rated health* was evaluated by the question ‘In general, how would you rate your health today?’ with answer options of very good, good, moderate, bad, and very bad. Those who answered bad or very bad were considered to have poor self-rated health. Self-rated health has been reported to be a valid indicator of overall health and a strong predictor of outcomes such as mortality^15^. *Smoking* was assessed by the question ‘Do you currently smoke any tobacco products such as cigarettes, cigars, or pipes?’ with the answer options being ‘daily’, ‘yes, but not daily’, or ‘no, not at all’. *Alcohol consumption* was assessed by first asking the respondent ‘Have you ever consumed a drink that contained alcohol (such as beer, wine, etc)?’ Those who answered negatively were considered lifetime abstainers. Those who answered affirmatively were then asked how many standard drinks of any alcoholic beverage they had on each of the past 7 days. The number of days on which 4 (female) or 5 (male) drinks were consumed was then calculated^16^, and 1–2 and >3 days in the past 7 days were considered infrequent and frequent heavy drinking respectively. Those not categorized as either infrequent or frequent heavy drinkers were grouped as lifetime abstainers or non-heavy drinkers. The prevalence of a subset of *chronic conditions*, including angina, arthritis, asthma, and diabetes, was based on a lifetime self-reported diagnosis. For angina, in addition to self-report, a symptom-based diagnosis based on the Rose questionnaire was also used^17^. Qualitative information on *disability* was collected by asking how much difficulty each respondent had in the past 30 days in moving around, performing self-care (such as washing or dressing), concentrating or remembering things, and seeing and recognizing a person across the road. Answer options were none, mild, moderate, severe, and extreme. Those who answered severe or extreme to any of these questions were categorized as having disability^18^.

### Statistical analysis

All analyses were done with Stata statistical software version 13.1 (Stata Corp LP, College Station, Texas). Data were publicly available for 69 countries. Countries with ≥25% of missing data on edentulism (9 countries–Congo, Ecuador, Mali, Mauritania, Mexico, Slovakia, Swaziland, Tunisia, United Arab Emirates) and no sampling information (10 countries–Austria, Belgium, Denmark, Germany, Greece, Guatemala, Italy, Netherlands, Slovenia, and UK) were excluded from the analysis. Based on the World Bank classification in 2003, the remaining 50 countries (n = 201,953) consisted of 9 high- (n = 14,658), 23 middle- (n = 93,872), and 18 low-income (n = 93,423) countries. The prevalence of edentulism was calculated for all of these 50 countries including HICs for global comparison. We adjusted for different age and sex structures of each country by standardizing the prevalence estimates using United Nations population pyramids for the year 2010 (http://esa.un.org/wpp/Excel-Data/population.htm) as the reference population.

For subsequent analyses, HICs were excluded because the focus of this paper was on LMICs. Furthermore, Turkey was also excluded, as information on some variables used for the analysis were not collected. This resulted in a total of 40 LMICs being included in the subsequent analyses (n = 175,814). We planned *a priori* an age-stratified analysis: <50 and ≥50 years. This was planned because of the different causes of edentulism in each age group where edentulism is considered to be primarily attributable to dental caries in younger patients while periodontitis is the primary cause in older persons, and for consistency with previous epidemiologic studies^5^,^19^,^20^. Furthermore, while there is extensive literature on edentulism in older ages, data on the younger population is scarce. Multivariable logistic regression analyses were used to assess the correlates of edentulism. The selection of the covariates used for adjustment was based on previous studies^3^,^21^. All models with edentulism as the outcome adjusted for sex, education, age, wealth, alcohol consumption, smoking, chronic conditions, and country. The analyses with depression and poor self-rated health as the outcomes adjusted for the same above-mentioned covariates as well as disability. With the exception of age, all covariates were categorical variables. A sensitivity analysis was performed with age as a categorical variable based on cut-offs of 50 years, and the median age of age groups <50 years and ≥50 years. Taylor linearization methods accounted for sample weighting schemes and complex study design. Results from the logistic regression models are presented as odds ratios (ORs) with their 95% confidence intervals (CIs). The level of statistical significance was set at P<0.05.

Less than 5% of the data for the variables used in the regression analysis were missing with the exception of wealth (9.1%), edentulism (5.9%), diabetes (5.7%), and arthritis (5.3%). We conducted multiple imputation procedures (10 additional samples) using Stata’s ICE program to assess whether the presence of these missing values led to biased estimates^22^. Since the results of the analysis with and without imputed data were similar, we only present non-imputed results.

## Results

**Figure 1:**
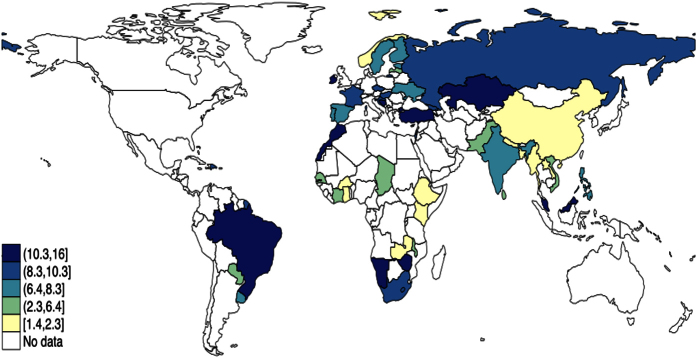
Age-sex adjusted prevalence of edentulism. All age-sex adjusted weighed estimates were calculated using the United Nations population pyramids for the year 2010. Data from 50 countries are presented. The figure was created with STATA 13.1 (StataCorp. 2013. *Stata Statistical Software: Release 13*. College Station, TX: StataCorp LP).

The mean age of the sample was 39.1 years. The overall age and sex-standardized prevalence of edentulism was 7.6% [range: 1.4% (Bangladesh and Myanmar) to 15.2% (Brazil)] (Fig. 1). The corresponding figure for those <50 and ≥50 years was 2.8% [range: 0.1% (Myanmar) to 14.5% (Zimbabwe)] and 14.0% [range: 2.1% (Ghana) to 32.3% (Brazil)] respectively. Prevalence estimates with 95% CIs for all WHS countries are presented in Table 1. The prevalence overall tended to be lower in low-income countries (LICs). The sample characteristics of the 40 LMICs by age group are shown in Table 2. Within the subset of those >50 years old, female sex, age, lower education, daily smoking, and some chronic conditions (arthritis, asthma, and diabetes) were significantly associated with higher odds for edentulism. The only significant correlates in those <50 years were female sex and age (Table 3). After adjustment for these potential confounders, edentulism was associated with a 1.57 (95% CI 1.23–2.00) times higher odds for depression among those aged <50 years (Table 4). However, this was not significant in the older age group [OR 1.03 (95% CI 0.84–1.26)]. Similarly, edentulism was associated with 1.38 (95% CI 1.03–1.83) times higher odds for poor self-rated health in the younger age group but not in the older age group [OR 1.04 (95% CI 0.89–1.23)]. In sensitivity analyses with age as a categorical variable, results were quantitatively similar (eTable1, eTable2). Edentulism was associated with depression for all age groups, except for persons ≥61 years OR 0.86 (95% CI 0.67–1.12) (eTable 2). Odds ratios for edentulism and self-rated health were quantitatively similar to the main analyses and showed a trend for association in the 31–49 years and 50–60 years age groups (eTable 2).

## Discussion

The present work revealed that edentulism is a highly prevalent condition globally with an overall age- and sex-standardized prevalence of 7.6% ranging from as low as 1.4% in Bangladesh and Myanmar up to 15.2% in Brazil. The global prevalence figures among those <50 and ≥50 years were 2.8% and 14.0% respectively. Our analysis supported the association between most previously reported risk factors and edentulism, although some were not statistically significant in those <50 years old. Age and female gender were significantly correlated in both older and younger age groups. In those ≥50 years old, lower education, smoking, arthritis, asthma, diabetes were also significantly associated with edentulism. Our data also showed that edentulism is associated with depression and poor self-rated health in those <50 years old. To the best of our knowledge, this is the first large-scale multi-continent study on edentulism using mostly nationally representative data, which assessed the prevalence and correlates of edentulism and its association with depression and self-rated health.

Age-sex standardized prevalence of edentulism was highest in MICs (10.9%), followed by HICs (8.6%), and LICs (4.4%). The overall prevalence of edentulism in those <50 years was 2.8% (LICs 1.6%; MICs 4.3%; HICs 3.5%) with the highest prevalence observed in Zimbabwe (14.5%), Nambia (13.2%), and South Africa (8.2%). Countries with high overall edentulism prevalence rates also tended to have higher rates in the younger age group. Only a limited number of published studies have reported on the trans-national prevalence of edentulism in older individuals^3^,^10^,^21^ and only one study has reported rates of edentulism in younger persons^23^. Despite limitations in comparability due to methodological differences, the prevalence of edentulism observed in our study falls within the range of each of these reports. Consistency with GBD 2010^23^, which analyzed literature and national report data to generate prevalence and burden estimates, lends further support to the finding that edentulism is non-trivial even in younger populations, and that prevention efforts targeting oral health in younger persons need to be expanded in many LMICs.

The low prevalence in LICs may be related to low consumption of non-refined carbohydrates and the resulting low prevalence of dental caries^24^. The large variation observed within countries at similar income-levels suggests that average per capita income may not be a primary determinant of edentulism. Other factors such as oral hygiene practices, nutritional habits, and socioeconomic inequalities have been suggested as being stronger driving factors^3^. Our analysis did not find an independent association between wealth quintile and edentulism, which may at first glance contradict this suggestion, but it accords with a recent study from Ghana^25^. Furthermore, it aligns with what has been proposed by other researchers^26^, namely that the socioeconomic inequality with respect to edentulism is not captured solely by measurement of wealth, but rather it also reflects oral health education such as better dental health awareness, dentist attitudes, and oral health care philosophies as well as access to oral health care centers.

We found a significant association between edentulism and depression and poor self-rated health in the younger age group (<50 years). This finding highlights the potentially deleterious physical and social repercussions of edentulism in younger ages. Previous research has documented the effect of edentulism on facial appearance, speech, and eating that may have an impact on mental health and wellbeing through consequences such as low self-esteem, and decline in social activities due to embarrassment^27^,^28^. When this occurs at an age when edentulism is uncommon, the relative impact may be larger. Furthermore, the thought of having to live with edentulism forever might lead to more hopelessness and depression in younger individuals.

There are several limitations to this study. First, the cross-sectional design of WHS data collection limits the potential for etiological conclusions. Second, residual confounding may exist since we could not adjust for factors such as dietary habits, social cohesion, access to dental care, and some other comorbidities (e.g. stroke, memory problems, lung disease, etc) due to lack of data. Thus, their confounding and individual effects remain unknown. Third, records of dental caries and periodontal disease were not available either, precluding the possibility to reveal the underlying causes of edentulism. In addition, since all information was based on self-report, reporting bias may have existed. For example, although self-report of diseases (e.g. diabetes) has been shown to demonstrate good agreement with medical records in developed countries, in settings with limited access to health care, under-reporting of chronic conditions is likely to have occurred^29^. Social-desirability bias in self-reported tobacco and alcohol consumption data may similarly have led to an underestimation of their correlation with edentulism^30^. Finally, the countries included in the WHS were not selected in a probabilistic manner to be representative of the world but have rather been chosen based on convenience. Thus, it is possible that our estimates are not representative of all LMICs.

While there are several studies on edentulism and quality of life^25^, to date, there has been little evidence regarding its association with depression and poor self-rated health, and until now there has been no evidence at all in LMICs. Determining the drivers of the phenomenon we have observed should be an area of further research. Is the positive correlation between edentulism, depression and poor self-rated health due to stronger social stigmas in LMIC, limited or sub-optimal treatment and adaptive equipment options, or because of other currently unquantified factors? Parsing the answer to this question will be crucial for evaluating strategies to limit the health consequences of edentulism in LMIC.

In conclusion, edentulism is a highly prevalent condition globally and it seems to be a common problem among LMIC. Age-sex standardized estimates ranged from 0.1% to 32.3% among the analyzed WHS countries. Among the factors related with edentulism, age and female gender were common in both older and younger age groups while socio-demographic factors (i.e., lower education), lifestyle habits (i.e., smoking habits), and health conditions (i.e., arthritis, asthma and diabetes) were related with edentulism only in the older age group. Edentulism was associated with depression and poor self-rated health only in the younger age group.

As we are now living in an era when aging and increased life expectancy are expected to progress, early-onset edentulism and its associated sequelae have the potential to become a growing public health problem. Relatedly, the finding that edentulism is associated with other NCDs and that they share common risk factors means that efforts to limit exposure to those common risk factors may also lead to reduction in edentulism burden. Both of these factors, coupled with the relative ease by which edentulism can be accurately measured and monitored, support full integration of edentulism surveillance and control into the global agenda of controlling NCDs.

## Additional Information

**How to cite this article**: Tyrovolas, S. *et al.* Population prevalence of edentulism and its association with depression and self-rated health. *Sci. Rep.* **6**, 37083; doi: 10.1038/srep37083 (2016).

**Publisher’s note:** Springer Nature remains neutral with regard to jurisdictional claims in published maps and institutional affiliations.

## Supplementary Material

Supplementary Information

## Figures and Tables

**Table 1:** Age-sex adjusted prevalence (95% CI) of edentulism by country and age group.

Country	N^a^	Overall	Age <50 years	Age ≥50 years
High-income countries				
Finland	1,013	8.3 (6.7–10.3)	3.0 (1.3–6.9)	13.4 (10.9–16.4)
France	1,008	9.8 (7.0–13.6)	4.1 (2.1–7.8)	15.7 (10.9–22.0)
Ireland	1,014	14.3 (11.8–17.2)	5.7 (3.4–9.2)	22.8 (17.9–28.6)
Israel	1,536	9.8 (8.2–11.8)	3.1 (1.9–4.9)	17.4 (13.3–22.4)
Luxembourg	700	10.0 (8.0–12.5)	8.1 (5.9–11.0)	11.2 (7.7–16.1)
Norway	984	2.1 (1.4–3.2)	0.3 (0.1–1.4)	4.7 (2.8–7.7)
Portugal	1,030	7.9 (6.5–9.6)	2.0 (0.9–4.1)	15.0 (11.0–20.2)
Spain	6,373	7.6 (6.8–8.5)	2.8 (1.8–4.2)	12.1 (10.7–13.7)
Sweden	1,000	5.1 (3.4–12.5)	4.9 (1.8–12.5)	5.4 (2.9–9.7)
Total	14,658	8.6 (7.3–10.2)	3.5 (2.4–5.0)	13.6 (11.4–16.2)
Middle-income countries				
Bosnia Herzegovina	1,031	14.0 (11.8–16.6)	3.9 (2.2–6.6)	27.9 (22.5–34.1)
Brazil	5,000	15.2 (14.0–16.5)	5.2 (4.3–6.2)	32.3 (29.0–35.8)
China	3,994	2.3 (1.8–2.9)	0.2 (0.0–0.9)	3.8 (2.9–4.9)
Croatia	993	8.5 (6.9–10.5)	3.0 (1.4–6.3)	14.4 (11.3–18.2)
Czech Republic	949	8.9 (7.0–11.1)	2.3 (1.0–4.9)	16.1 (12.0–21.3)
Dominican Republic	5,027	9.3 (8.2–10.5)	1.9 (1.3–2.6)	18.3 (15.4–21.5)
Estonia	1,020	8.2 (6.6–10.0)	2.9 (1.8–4.6)	14.5 (10.9–19.1)
Georgia	2,950	9.3 (7.8–11.1)	2.4 (1.5–3.8)	16.5 (13.0–20.8)
Hungary	1,419	9.8 (8.4–11.4)	1.7 (0.9–3.2)	19.3 (15.9–23.2)
Kazakhstan	4,499	12.0 (8.0–17.5)	6.1 (3.4–10.6)	19.9 (13.3–28.6)
Latvia	929	5.1 (3.9–6.6)	0.5 (0.1–2.0)	9.4 (6.7–13.1)
Malaysia	6,145	10.6 (9.7–11.6)	2.9 (2.3–3.6)	22.2 (19.8–24.8)
Mauritius	3,968	13.6 (11.9–15.5)	4.5 (3.0–6.7)	27.1 (23.7–30.7)
Morocco	5,000	10.8 (9.5–12.2)	4.2 (3.1–5.8)	20.6 (17.5–24.2)
Namibia	4,379	16.0 (14.2–17.9)	13.2 (11.3–15.4)	20.5 (16.5–25.2)
Paraguay	5,288	5.2 (4.5–6.0)	2.0 (1.5–2.7)	10.4 (8.5–12.5)
Philippines	10,083	7.7 (6.9–8.7)	3.5 (2.8–4.5)	14.6 (12.7–16.7)
Russia	4,427	8.7 (7.2–10.4)	3.6 (2.4–5.3)	14.9 (12.0–18.3)
South Africa	2,629	10.3 (8.1–13.0)	8.2 (6.0–11.2)	15.3 (10.6–21.4)
Sri Lanka	6,805	5.1 (4.3–6.0)	1.2 (0.8–1.8)	8.9 (7.3–10.9)
Turkey	11,481	13.9 (12.9–14.9)	5.0 (4.2–5.8)	28.2 (25.7–30.8)
Ukraine	2,860	7.3 (5.7–9.3)	2.1 (1.1–3.9)	14.5 (10.3–19.9)
Uruguay	2,996	6.8 (6.0–7.6)	1.1 (0.8–1.5)	14.0 (12.4–15.8)
Total	93,872	10.9 (10.4–11.4)	4.3 (3.9–4.7)	21.2 (20.0–22.5)
Low-income countries				
Bangladesh	5,942	1.4 (1.0–2.0)	0.2 (0.1–0.5)	3.0 (2.1–4.3)
Burkina Faso	4,948	2.0 (1.4–2.9)	0.5 (0.2–0.9)	4.1 (2.5–6.5)
Chad	4,870	6.4 (5.1–8.1)	3.5 (2.5–4.8)	11.0 (8.2–14.6)
Comoros	1,836	3.0 (2.1–4.2)	1.8 (0.9–3.6)	5.0 (3.1–8.0)
Ivory Coast	3,251	5.5 (4.0–7.4)	4.9 (3.6–6.7)	6.1 (3.5–10.5)
Ethiopia	5,089	1.6 (1.1–2.4)	0.6 (0.3–1.1)	3.4 (2.1–5.4)
Ghana	4,165	1.8 (1.3–2.3)	0.7 (0.4–1.1)	2.1 (1.5–3.0)
India	10,687	7.5 (5.9–9.4)	3.0 (2.1–4.3)	13.0 (10.0–16.8)
Kenya	4,640	1.5 (1.0–2.1)	0.5 (0.2–1.0)	2.7 (1.6–4.4)
Laos	4,988	2.3 (1.8–2.9)	0.6 (0.4–1.1)	4.2 (3.1–5.5)
Malawi	5,551	2.9 (2.3–3.6)	2.3 (1.7–3.0)	3.1 (2.1–4.7)
Myanmar	6,045	1.4 (1.1–1.9)	0.1 (0.0–0.3)	2.4 (1.8–3.3)
Nepal	8,820	1.9 (1.5–2.3)	0.5 (0.4–0.8)	3.3 (2.4–4.5)
Pakistan	6,501	6.0 (5.0–7.3)	1.9 (1.1–3.2)	10.1 (8.3–12.2)
Senegal	3,461	5.9 (4.7–7.3)	4.2 (3.1–5.7)	9.7 (6.7–14.0)
Vietnam	4,174	3.2 (2.3–4.6)	0.6 (0.2–1.6)	5.9 (3.9–8.8)
Zambia	4,165	2.3 (1.5–3.5)	2.0 (1.1–3.4)	3.4 (1.8–6.6)
Zimbabwe	4,290	13.0 (11.6–14.5)	14.5 (13.1–15.9)	6.7 (4.7–9.6)
Total	93,423	4.4 (4.0–4.9)	1.6 (1.3–1.9)	7.6 (6.7–8.6)

Data are weighted % (95% CI) unless otherwise stated.

All age-sex adjusted weighted estimates were calculated using the United Nations population pyramids for the year 2010.

^a^Unweighted N.

**Table 2:** Characteristics of the study sample.

Characteristic	Category	Age <50 years		Age ≥50 years	
		N^a^	%	N^a^	%
Sex	Male	56,026	50.4	20,363	46.0
	Female	67,873	49.6	25,386	54.0
Education	No formal	27,016	24.0	16,704	40.7
	≤Primary	46,691	33.0	15,889	29.6
	Secondary completed	37,914	32.8	9,319	21.0
	Tertiary completed	12,144	10.2	3,792	8.7
Wealth	Poorest	25,863	19.3	12,262	22.5
	Poorer	23,188	19.5	9,498	21.4
	Middle	22,459	20.1	7,717	19.4
	Richer	22,180	20.5	6,818	18.4
	Richest	21,588	20.6	6,222	18.3
Alcohol consumption	Never or non-heavy	114,652	94.7	43,168	96.5
	Infrequent heavy	5,712	4.2	1,304	2.5
	Frequent heavy	1,645	1.1	588	1.0
Smoking	None	93,751	74.3	33,063	70.1
	Not daily	6,408	5.1	2,200	4.5
	Daily	22,782	20.6	10,120	25.4
Arthritis	No	110,500	91.1	32,665	71.5
	Yes	11,374	8.9	11,940	28.5
Angina	No	107,703	88.4	33,728	72.9
	Yes	14,970	11.6	11,403	27.1
Asthma	No	116,986	95.6	41,788	91.8
	Yes	4,997	4.4	3,160	8.2
Diabetes	No	119,512	98.6	41,482	92.3
	Yes	1,636	1.4	3,068	7.7
Disability	No	113,271	91.0	33,378	70.9
	Yes	10,050	9.0	12,184	29.1
Depression	No	111,049	89.7	38,944	84.5
	Yes	11,584	10.3	6,211	15.5
Self-rated health	Not poor	116,209	93.9	36,793	78.6
	Poor	6,913	6.1	8,693	21.4

Data are % based on weighted sample.

A total of 40 low- and middle-income countries are included in the analysis.

^a^Unweighed N.

**Table 3:** Correlates of edentulism by age group estimated with multivariable logistic regression.

Characteristics	Age <50 years			Age ≥50 years		
	OR	95% CI	P-value	OR	95% CI	P-value
Sex						
Male	1.00			1.00		
Female	1.33	(1.12–1.59)	0.001	1.46	(1.29–1.66)	<0.001
Age (years)	1.05	(1.04–1.06)	<0.001	1.08	(1.07–1.08)	<0.001
Education						
No formal	1.00			1.00		
≤Primary	1.13	(0.87–1.46)	0.359	1.02	(0.86–1.21)	0.801
Secondary completed	0.84	(0.63–1.13)	0.261	0.72	(0.57–0.91)	0.006
Tertiary completed	0.69	(0.47–1.01)	0.059	0.43	(0.32–0.58)	<0.001
Wealth						
Poorest	0.93	(0.73–1.20)	0.586	1.12	(0.93–1.35)	0.245
Poorer	1.18	(0.92–1.53)	0.199	1.17	(0.98–1.41)	0.088
Middle	1.00			1.00		
Richer	0.99	(0.74–1.32)	0.930	1.13	(0.92–1.39)	0.244
Richest	0.96	(0.73–1.26)	0.784	1.03	(0.83–1.29)	0.760
Alcohol consumption						
Never or non-heavy	1.00			1.00		
Infrequent heavy	0.80	(0.54–1.19)	0.279	0.76	(0.51–1.13)	0.179
Frequent heavy	1.26	(0.69–2.30)	0.448	1.23	(0.73–2.06)	0.442
Smoking						
None	1.00			1.00		
Not daily	1.21	(0.82–1.77)	0.335	1.21	(0.92–1.59)	0.174
Daily	1.19	(0.97–1.46)	0.102	1.28	(1.09–1.49)	0.002
Chronic conditions						
Arthritis	1.37	(0.99–1.89)	0.058	1.23	(1.06–1.42)	0.006
Angina	1.12	(0.90–1.39)	0.321	1.06	(0.92–1.22)	0.403
Asthma	1.31	(0.99–1.72)	0.055	1.27	(1.03–1.55)	0.023
Diabetes	1.10	(0.72–1.69)	0.650	1.33	(1.09–1.62)	0.005

Abbreviation: OR odds ratio; CI confidence interval.

A total of 40 low- and middle-income countries are included in the analysis.

Models are mutually adjusted for all covariates in the table and country.

**Table 4:** The association of edentulism with depression and poor self-rated health by age group estimated with multivariable logistic regression.

Characteristic	Depression				Poor self-rated health			
	Age < 50		Age ≥ 50		Age < 50		Age ≥ 50	
	OR*	P-value	OR*	P-value	OR*	P-value	OR*	P-value
Edentulism	1.57	0.003	1.03	0.789	1.38	0.028	1.04	0.605
	(1.23–2.00)		(0.84–1.26)		(1.03–1.83)		(0.89–1.23)	
Sex								
Male	1.00		1.00		1.00		1.00	
Female	1.64	<0.001	1.63	<0.001	1.30	0.004	1.24	0.001
	(1.46–1.84)		(1.43–1.85)		(1.09–1.55)		(1.09–1.41)	
Age (years)	1.01	<0.001	0.99	0.051	1.03	<0.001	1.02	<0.001
	(1.01–1.02)		(0.99–1.00)		(1.02–1.03)		(1.01–1.02)	
Education								
No formal	1.00		1.00		1.00		1.00	
≤Primary	1.12	0.092	1.07	0.434	0.96	0.614	0.90	0.2127
	(0.98–1.27)		(0.90–1.27)		(0.83–1.12)		(0.76–1.07)	
Secondary completed	1.17	0.029	1.10	0.427	0.72	0.004	0.72	0.025
	(1.02–1.36)		(0.87–1.39)		(0.58–0.90)		(0.55–0.96)	
Tertiary completed	1.27	0.212	1.40	0.026	0.75	0.385	0.51	<0.001
	(0.87–1.86)		(1.04–1.88)		(0.39–1.44)		(0.37–0.70)	
Wealth								
Poorest	0.88	0.039	1.04	0.673	1.26	0.005	1.26	0.009
	(0.77–0.99)		(0.86–1.26)		(1.07–1.48)		(1.06–1.50)	
Poorer	0.94	0.384	1.00	0.985	1.11	0.22	1.24	0.019
	(0.82–1.08)		(0.84–1.19)		(0.94–1.30)		(1.03–1.48)	
Middle	1.00		1.00		1.00		1.00	
Richer	0.94	0.467	1.07	0.519	1.03	0.844	0.97	0.784
	(0.80–1.11)		(0.88–1.29)		(0.77–1.37)		(0.79–1.19)	
Richest	0.89	0.168	0.99	0.948	0.75	0.022	0.67	0.001
	(0.75–1.05)		(0.78–1.26)		(0.59–0.96)		(0.52–0.85)	
Alcohol consumption								
Never or non-heavy	1.00		1.00		1.00		1.00	
Infrequent heavy	0.93	0.504	0.74	0.131	0.91	0.619	0.80	0.309
	(0.75–1.15)		(0.50–1.09)		(0.63–1.31)		(0.53–1.22)	
Frequent heavy	1.10	0.633	0.91	0.787	0.93	0.763	1.01	0.962
	(0.75–1.59)		(0.48–1.74)		(0.60–1.46)		(0.64–1.61)	
Smoking								
No	1.00		1.00		1.00		1.00	
Not daily	1.25	0.048	1.05	0.774	0.92	0.545	1.12	0.436
	(1.00–1.55)		(0.75–1.47)		(0.69–1.22)		(0.85–1.47)	
Daily	1.32	<0.001	1.17	0.044	1.03	0.724	1.13	0.132
	(1.17–1.50)		(1.01–1.37)		(0.87–1.23)		(0.96–1.32)	
Disability	2.61	<0.001	2.24	<0.001	5.45	<0.001	5.89	<0.001
	(2.32–2.93)		(1.91–2.64)		(4.80–6.19)		(5.21–6.66)	

^*^ORs and (95% Confidence Intervals are presented). Models are mutually adjusted for all covariates in the table and chronic conditions and country.
