# Supplementary material for: Population prevalence of edentulism and its association with depression and self-rated health
Source: Sci Rep. 2016 Nov 17;6:37083. doi: 10.1038/srep37083 (PMC5112530; doi:10.1038/srep37083)
Supplement: Supplementary Information [file srep37083-s1.doc]

**Population prevalence of edentulism and its association with depression and self-rated health**

Stefanos Tyrovolas^1,2^, Ai Koyanagi^1,2^, Demosthenes Panagiotakos^3^, Josep Maria Haro^1,2^, Nicholas J. Kassebaum^4^, Vanessa Chrepa^5^, Georgios A. Kotsakis^6^

^1^Parc Sanitari Sant Joan de Déu, Universitat de Barcelona. Fundació Sant Joan de Déu, Dr Antoni Pujades, 42, Sant Boi de Llobregat, 08830, Barcelona, Spain

^2^Instituto de Salud Carlos III, Centro de Investigación Biomédica en Red de Salud Mental, CIBERSAM, Monforte de Lemos 3-5, Pabellón 11, 28029, Madrid, Spain

^3^Department of Nutrition and Dietetics, School of Health Science and Education, Harokopio University, Athens, Greece

^4^Institute for Health Metrics and Evaluation & Department of Anesthesiology & Pain Medicine, Seattle Children’s Hospital, University of Washington, Seattle, WA, USA

^5^Department of Endodontics, University of Washington, Seattle, WA, USA

^6^Department of Periodontics, University of Washington, Seattle, WA, USA

**Address for Correspondence**

Stefanos Tyrovolas, PhD

Parc Sanitari Sant Joan de Déu, Fundació Sant Joan de Déu, CIBERSAM,

Dr. Antoni Pujadas, 42, 08830 – SantBoi de Llobregat, Barcelona, Spain.

Email: [s.tyrovolas@pssjd.org](mailto:s.tyrovolas@pssjd.org)

Georgios A Kotsakis, DDS, MS

Department of Periodontics, University of Washington

1959 NE Pacific St, Box# 357444, Seattle, WA 98195-7444

Email: kotsakis@uw.edu

| **eTable 1** Correlates of edentulism by four age groups estimated with multivariable logistic regression | | | | | | | | |
| --- | --- | --- | --- | --- | --- | --- | --- | --- |
|  | Age <50 years | | | | Age ≥50 years | | | |
|  | 18-30 years | | 31-49 years | | 50-60 years | | ≥61 years | |
| Characteristic | OR [95%CI] | P-value | OR [95%CI] | P-value | OR [95%CI] | P-value | OR [95%CI] | P-value |
| **Sex** |  |  |  |  |  |  |  |  |
| Male | 1.00 |  | 1.00 |  | 1.00 |  | 1.00 |  |
| Female | 1.13 | 0.449 | 1.49 | <0.001 | 1.40 | 0.001 | 1.50 | <0.001 |
|  | [0.82,1.56] |  | [1.22,1.83] |  | [1.15,1.72] |  | [1.28,1.76] |  |
| **Age** (years) | 1.04 | 0.029 | 1.08 | <0.001 | 1.10 | <0.001 | 1.07 | <0.001 |
|  | [1.00,1.09] |  | [1.06,1.10] |  | [1.07,1.13] |  | [1.06,1.08] |  |
| **Education** |  |  |  |  |  |  |  |  |
| No formal | 1.00 |  | 1.00 |  | 1.00 |  | 1.00 |  |
| ≤Primary | 1.04 | 0.875 | 1.19 | 0.276 | 0.98 | 0.858 | 1.05 | 0.695 |
|  | [0.66,1.62] |  | [0.87,1.61] |  | [0.74,1.28] |  | [0.84,1.31] |  |
| Secondary completed | 0.91 | 0.700 | 0.80 | 0.217 | 0.59 | 0.006 | 0.82 | 0.169 |
|  | [0.55,1.50] |  | [0.56,1.14] |  | [0.40,0.86] |  | [0.62,1.09] |  |
| Tertiary completed | 0.80 | 0.500 | 0.65 | 0.078 | 0.45 | 0.001 | 0.43 | <0.001 |
|  | [0.42,1.53] |  | [0.40,1.05] |  | [0.28,0.72] |  | [0.30,0.62] |  |
| **Wealth** |  |  |  |  |  |  |  |  |
| Poorest | 0.90 | 0.648 | 0.97 | 0.821 | 1.17 | 0.286 | 1.09 | 0.469 |
|  | [0.58,1.40] |  | [0.72,1.30] |  | [0.88,1.57] |  | [0.87,1.36] |  |
| Poorer | 0.92 | 0.707 | 1.36 | 0.038 | 1.27 | 0.090 | 1.12 | 0.325 |
|  | [0.60,1.41] |  | [1.02,1.81] |  | [0.96,1.67] |  | [0.89,1.41] |  |
| Middle | 1.00 |  | 1.00 |  | 1.00 |  | 1.00 |  |
| Richer | 1.14 | 0.661 | 0.90 | 0.484 | 1.19 | 0.251 | 1.12 | 0.415 |
|  | [0.64,2.04] |  | [0.67,1.21] |  | [0.88,1.61] |  | [0.86,1.45] |  |
| Richest | 1.07 | 0.760 | 0.89 | 0.506 | 1.02 | 0.904 | 1.05 | 0.763 |
|  | [0.68,1.71] |  | [0.64,1.25] |  | [0.74,1.41] |  | [0.78,1.40] |  |
| **Alcohol consumption** |  |  |  |  |  |  |  |  |
| Never or non-heavy | 1.00 |  | 1.00 |  | 1.00 |  | 1.00 |  |
| Infrequent heavy | 0.87 | 0.718 | 0.81 | 0.372 | 0.71 | 0.188 | 0.79 | 0.435 |
|  | [0.42,1.83] |  | [0.50,1.30] |  | [0.43,1.18] |  | [0.43,1.43] |  |
| Frequent heavy | 1.37 | 0.553 | 1.26 | 0.488 | 0.97 | 0.938 | 1.34 | 0.413 |
|  | [0.48,3.92] |  | [0.66,2.39] |  | [0.45,2.10] |  | [0.67,2.68] |  |
| **Smoking** |  |  |  |  |  |  |  |  |
| None | 1.00 |  | 1.00 |  | 1.00 |  | 1.00 |  |
| Not daily | 0.69 | 0.122 | 1.55 | 0.059 | 0.86 | 0.494 | 1.48 | 0.040 |
|  | [0.42,1.11] |  | [0.98,2.45] |  | [0.56,1.32] |  | [1.02,2.14] |  |
| Daily | 1.15 | 0.483 | 1.21 | 0.114 | 1.29 | 0.029 | 1.25 | 0.041 |
|  | [0.77,1.72] |  | [0.95,1.54] |  | [1.03,1.61] |  | [1.01,1.56] |  |
| **Chronic conditions** |  |  |  |  |  |  |  |  |
| Arthritis | 1.41 | 0.111 | 1.32 | 0.160 | 1.18 | 0.162 | 1.25 | 0.008 |
|  | [0.92,2.14] |  | [0.90,1.95] |  | [0.94,1.49] |  | [1.06,1.48] |  |
| Angina | 1.06 | 0.766 | 1.14 | 0.342 | 1.20 | 0.104 | 0.97 | 0.761 |
|  | [0.73,1.54] |  | [0.87,1.48] |  | [0.96,1.49] |  | [0.81,1.16] |  |
| Asthma | 1.57 | 0.164 | 1.22 | 0.207 | 1.39 | 0.060 | 1.22 | 0.103 |
|  | [0.83,2.95] |  | [0.90,1.65] |  | [0.99,1.96] |  | [0.96,1.54] |  |
| Diabetes | 0.62 | 0.411 | 1.11 | 0.668 | 1.58 | 0.009 | 1.19 | 0.139 |
|  | [0.20,1.92] |  | [0.69,1.77] |  | [1.12,2.23] |  | [0.95,1.49] |  |

Abbreviation: OR odds ratio; CI confidence interval

Models are mutually adjusted for all covariates in the table and country.

The four age groups were based on cut-offs of 50 years, and the median age of age groups <50 years and ≥50 years.

| **eTable 2** The association of edentulism with depression and poor self-rated health by four age groups estimated with multivariable logistic regression | | | | | | | | |
| --- | --- | --- | --- | --- | --- | --- | --- | --- |
|  | Age <50 years | | | | Age ≥50 years | | | |
|  | 18-30 years | | 31-49 years | | 50-60 years | | ≥61 years | |
| Outcome | OR [95%CI] | P-value | OR [95%CI] | P-value | OR [95%CI] | P-value | OR [95%CI] | P-value |
| Depression | 1.66 | 0.027 | 1.53 | 0.002 | 1.43 | 0.006 | 0.86 | 0.258 |
|  | [1.06,2.61] |  | [1.16,2.02] |  | [1.11,1.85] |  | [0.67,1.12] |  |
| Self-rated health | 1.52 | 0.213 | 1.30 | 0.100 | 1.29 | 0.152 | 0.99 | 0.900 |
|  | [0.79,2.92] |  | [0.95,1.79] |  | [0.91,1.82] |  | [0.83,1.18] |  |

Abbreviation: OR odds ratio; CI confidence interval

Models are adjusted for sex, age, education, wealth, alcohol consumption, smoking, disability, chronic conditions (arthritis, angina, asthma, diabetes), and country.

The four age groups were based on cut-offs of 50 years, and the median age of age groups <50 years and ≥50 years
